# Legionnaires Disease Presenting as Diarrhea: A Case Report

**DOI:** 10.7759/cureus.10593

**Published:** 2020-09-22

**Authors:** Nimit Dalal, Pal Satyajit Singh Athwal, Biswaraj Tharu, Parth Shah, Love Shah

**Affiliations:** 1 Internal Medicine, Trumbull Regional Medical Center, Warren, USA; 2 Internal Medicine, Saraswathi Institute of Medical Sciences, Hapur, IND

**Keywords:** legionnaires disease, diarrhea, legionella

## Abstract

Legionnaires disease is primarily a pneumonic illness with possible multisystem involvement. Major risk factors include immunodeficiency, smoking, alcoholism and chronic obstructive pulmonary disease among others. We report a peculiar case of Legionnaires disease presenting with diarrhea as the chief complaint and no respiratory symptoms throughout the course of disease. The patient had no risk factors for the disease and had no recent travel history or sick contacts. Acute diarrhea is not an uncommon manifestation of Legionnaires disease, although isolated diarrhea symptoms with the absence of concurrent respiratory symptoms and no risk factors for Legionella makes this case a diagnostic challenge, leading to possible delay in appropriate management. We are presenting this case to inform physicians of the possibility of Legionnaires disease presenting as an isolated gastrointestinal involvement with no clinical symptoms of pneumonia at presentation.

## Introduction

Legionella pneumophila is gram negative bacteria known for Legionnaires disease and pontiac fever. Legionella is responsible for 2-9% of community acquired pneumonia and can lead to multiorgan involvement with very high mortality without antibiotic therapy [1]. In 1976, members of American legion got infected from a point source in the hotel they were staying in and this incident framed the nomenclature of this bacterium [2]. Transmission is through breathing mist containing bacteria from a contaminated source. It presents mainly with respiratory symptoms like cough, shortness of breath, fever and myalgia following few days of exposure. Isolated gastrointestinal symptoms are very rare and can lead to missed diagnosis. Diagnosis is established based on sputum culture and urine antigen. Treatment is based on antibiotics and supportive therapy. We report a case of Legionnaires disease with isolated gastrointestinal symptoms which can lead to a missed diagnosis and development of complications without treatment.

## Case presentation

A 61-year-old female with past medical history of asthma, hypertension, hypothyroidism and chronic back pain presented to the ED with chief complaint of watery diarrhea and fever for four days. She denied any blood or mucus in stool. She was severely dehydrated. She also reported loss of appetite. She denied nausea, vomiting, chills, shortness of breath, cough and abdominal pain. The maximum recorded temperature at home was 101°F. On examination in the ED, her temperature was 100.9°F, heart rate 115 bpm, blood pressure 161/65 mmHg, respiratory rate of 14, O_2 _saturation was 97. Respiratory examination and abdominal examination was normal. Her labs on admission are shown in Table 1.

**Table 1:** Lab values

Lab values	
WBC	10 x 10^9^/L
Neutrophils	75.70%
lymphocytes	18.20%
Hemoglobin	12.4 g/dL
Platelets	161,000
Sodium	136 mEq/L
Potassium	3 mEq/L
Creatinine	1.18
Glomerular filtration rate	50 mL/min

**Figure 1:**
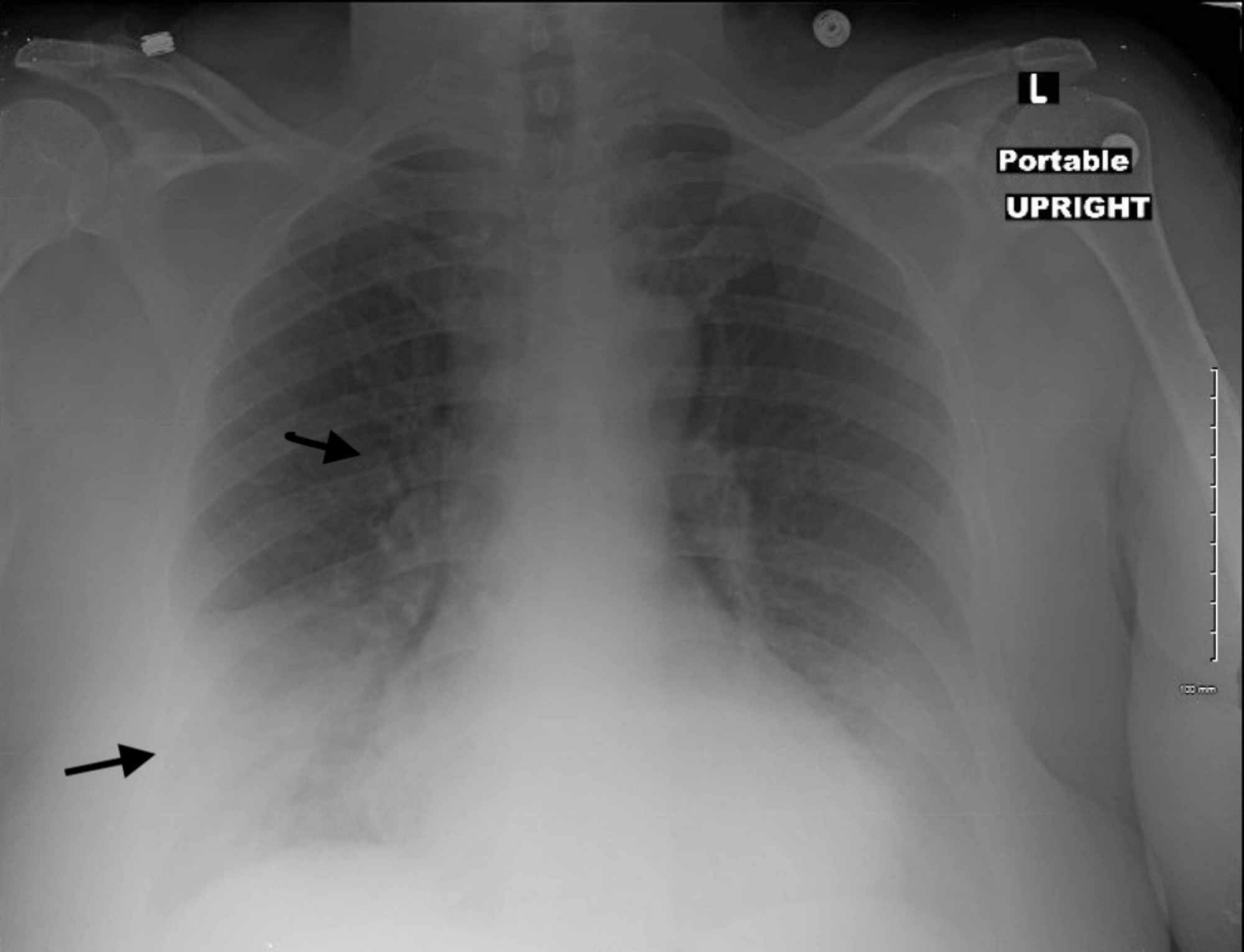
X-ray chest

She was initially treated with IV fluids for suspected gastroenteritis. Stool sample was negative for culture, gram staining, microscopy for ova/parasites, and occult blood. Chest CT and chest X-ray done on the same day as per routine hospital protocol showed enlarged hilar lymph nodes with right lower lobe infiltrates and air bronchograms as shown in Figure 1.

She was started on IV ceftriaxone and IV azithromycin. EKG revealed tachycardia with premature atrial contractions and QTC prolonged to 523 ms. Legionella urine antigen came positive the next day and ceftriaxone was discontinued. Infectious diseases team was consulted. After much discussion, a decision was made to continue azithromycin. Covid-19 polymerase chain reaction (PCR) was ordered which turned out to be negative. The patient was kept under observation for a total of three days. Repeat EKG was done on day 3 of Azithromycin as initial EKG showed prolonged QT interval. EKG showed sinus rhythm with QTc interval of 438 ms. She was clinically stable during the entire stay with no temperature spikes or episodes of diarrhea. She was discharged on azithromycin 250 mg for four more days making the total duration of azithromycin therapy seven days.

The patient denied contact with any sick person and also denied recent travel. She worked in the kitchen to cook meals for homeless people. Source of infection was not identified and no further cases were reported in our hospital during that period.

## Discussion

Legionella bacteria are gram-negative, intracellular bacteria that are important causes of community-acquired and nosocomial pneumonia. Legionella infections can be acquired sporadically or during outbreaks. Legionella bacteria are typically transmitted via inhalation aerosols from contaminated water or soil. No such exposure was found in our patient. The term legionellosis refers to any clinical syndrome associated with Legionella infection. The two most common syndromes associated with Legionella infection are:

● Legionnaires' disease, which refers to pneumonia caused by Legionella spp

● Pontiac fever, which is an acute, self-limited febrile illness that is typically acquired during outbreaks [3].

Pneumonia caused by Legionella is clinically and radiographically similar to other forms of pneumonia. Predominant symptoms include fever, cough, and shortness of breath. Symptoms typically arise two to 10 days after exposure to contaminated water or soil. Fever and fatigue often precede the onset of cough. Rales and/or other signs of consolidation can be present on physical examination. Radiographic findings are varied and nonspecific; however, the most common findings are patchy unilobar infiltrates, which can progress to consolidations [4]. Extra pulmonary symptoms are not uncommon and include nausea, vomiting, diarrhea, acute kidney injury and in rare cases myocarditis, acute disseminated encephalomyelitis and multiorgan failure [5].

Diagnosis can be established based on culture, direct fluorescent antibody test, urine antigen test, and serum antibodies [6]. Sensitivity and specificity of diagnostic modalities are listed in Table 2 though sensitivity and specificity for serum antibodies varies depending on method used [7,8].

**Table 2:** Diagnostic tests for Legionnaires disease

Diagnostic Tests for Legionnaires Disease		
Test	Sensitivity	Specificity
Urine Antigen	69 - 100%	99 - 100%
Culture	81%	99%
Direct fluorescent antigen	70%	99%

In our case Legionella was detected in urine and appropriate antibiotic treatment was initiated.

This patient presented to the hospital with diarrhea as the chief complaint and no respiratory symptoms at all. It is very common to rule out pneumonia in a patient with no overt respiratory symptoms. Chest X-ray done in the ER as a part of admission protocol showed right middle lobe infiltrates and pneumonia was ruled in. Treatment should be initiated as soon as diagnosis is established with macrolides. Empiric therapy should be considered either as monotherapy with a macrolide or combined with a β-lactam agent. Respiratory fluoroquinolones such as levofloxacin can also be used [9].

We hypothesize that the mechanism of secretory diarrhea in our case maybe attributed directly to the host’s cell mediated immune response when bacterium invades macrophages for multiplication. Immune cascade is activated by lipopolysaccharide, lipid A, component of its outer membrane. Lipid A directly stimulates TNF-alpha production, which causes massive cytokine release and is primarily responsible for chloride secretion and intestinal inflammation [10]. It is important that clinicians be aware of this extra pulmonary manifestation of legionella infection, especially in patients with no risk factors or pulmonary symptoms of this disease.

## Conclusions

Legionnaires' disease is caused by gram negative, intracellular bacteria known as Legionella. It is a multisystem involvement infection predominantly present with respiratory symptoms. Patients usually have history of travel or known exposure, both of these provide clue to Legionella infection. We report a case of Legionnaires' disease in 61-year-old female patient without any known history of travel, risk factors and pulmonary symptoms. Such scenario poses a major challenge to diagnosis Legionnaires' disease. The main goal of this case report is to familiarize physicians of the possibility of Legionnaires disease presenting as an isolated gastrointestinal involvement with no clinical symptoms of pneumonia at presentation.
